# The critical role of mode of action studies in kinetoplastid drug discovery

**DOI:** 10.3389/fddsv.2023.1185679

**Published:** 2023-05-10

**Authors:** Alan H. Fairlamb, Susan Wyllie

**Affiliations:** Wellcome Centre for Anti-Infectives Research, Division of Biological Chemistry and Drug Discovery, School of Life Sciences, University of Dundee, Dundee, United Kingdom

**Keywords:** whole genome sequencing, chemical proteomics, drug resistance, chemical pulldown, metabolomics, trypanosomiasis, leishmaniasis, target identification

## Abstract

Understanding the target and mode of action of compounds identified by phenotypic screening can greatly facilitate the process of drug discovery and development. Here, we outline the tools currently available for target identification against the neglected tropical diseases, human African trypanosomiasis, visceral leishmaniasis and Chagas’ disease. We provide examples how these tools can be used to identify and triage undesirable mechanisms, to identify potential toxic liabilities in patients and to manage a balanced portfolio of target-based campaigns. We review the primary targets of drugs that are currently in clinical development that were initially identified via phenotypic screening, and whose modes of action affect protein turnover, RNA trans-splicing or signalling in these protozoan parasites.

## 1 Introduction

The development of better and safer drugs to treat kinetoplastid diseases such as Leishmaniasis and Chagas’ disease and many other infectious diseases has been hampered by a severe lack of drug targets that have been robustly validated genetically (for essentiality) and chemically (for druggability) (Frearson et al., 2007; Wyatt et al., 2011; Field et al., 2017). This has left drug discovery programs heavily reliant upon whole cell (phenotypic) screening to identify suitable chemical start points. Development and optimisation of phenotypically active compounds is also hindered by lack of information regarding the molecular target (s) and their mechanism (s) of action (Ziegler et al., 2013; Vincent et al., 2022). Specifically, knowledge of the molecular target is often crucial in developing strategies to overcome issues such as improving potency and selectivity against the parasite of interest and reducing toxicity against the mammalian host (Wyatt et al., 2011). Once the target of a phenotypically active compound series has been identified, target- and structure-based drug discovery programmes can be initiated allowing optimisation of potency and selectivity over human orthologues or the identification of novel chemical starting points (Terstappen et al., 2007). Knowing the mode of action of a compound series can also be used to abandon non-productive or undesirable drug discovery campaigns. Thus, mode of action studies can effectively integrate these two, often disconnected, approaches to drug discovery. Furthermore, understanding the mode of action is helpful in selecting appropriate partner drugs for future combination therapies. Although not considered in any detail here, some of the approaches used here could be used to identify undesirable “off-target” causes of toxicity in the mammalian host (Schenone et al., 2013).

## 2 Tools for target identification and validation

Target identification and mode of action studies uses a wide range of technologies that investigate different features of a cell—ranging from gross morphological structure through to changes in DNA, RNA, protein, and lipid and energy metabolism.

### 2.1 Genomic approaches

**Figure 1:**
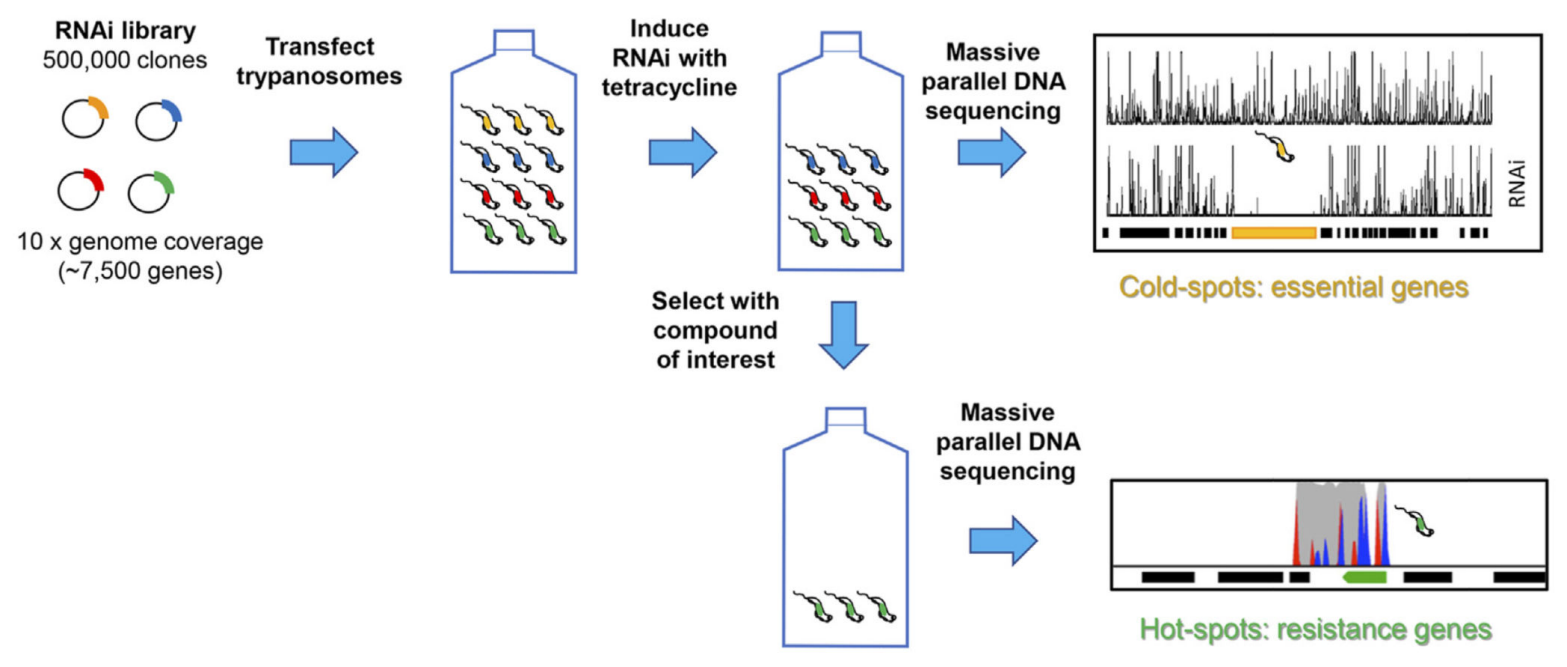
Schematic of RNAi approach to identifying essential genes and drug resistant mechanisms. A plasmid library containing randomly sheared genomic fragments was transfected into bloodstream form *T. brucei*. After culturing under non-inducing or inducing conditions, or in the presence or absence of drug, genomic DNA was isolated and adaptor-ligated libraries prepared. After amplification, size selection and sequencing, the RNAi target fragments are mapped to the reference genome. The depth of sequence coverage of target fragments between induced and uninduced cultures reveals “cold spots” (essential genes) and the depth of coverage of target fragments between induced drug treated and RNAi-induced controls reveals “hot spots” (resistance genes). Where gene knock-down confers a selective advantage. Full details are available elsewhere (Alsford et al., 2011; Alsford et al., 2012).

At the genomic level, there are three main unbiased approaches employed in target identification: target knockdown, target overexpression and whole genome sequencing of drug resistant cell lines (Begolo et al., 2014; Burle-Caldas Gde et al., 2015; Duncan et al., 2017). Target attenuation by knockdown of gene expression using RNA interference (RNAi) can only be applied to African trypanosomes since *T. cruzi* and *Leishmania* spp. (except *L. braziliensis*) lack the necessary RNAi machinery (Collett et al., 2019). Such genome-wide screens of inducible RNAi libraries have been used to identify genes that are tangential to drug action in *T. brucei* (Alsford et al., 2012), as well as essential genes (Alsford et al., 2011). As shown in Figure 1, replicating cells can only persist in the presence of a toxic compound if knockdown confers a selective advantage, so this method generally cannot identify the target *per se* (Fairlamb, 2012) Nonetheless, pathways and processes tangential to the target’s function can provide useful clues for subsequent investigations. Moreover, if the compound of interest that is active against *Leishmania* spp. or *T. cruzi* has similar potency against bloodstream form *T. brucei* then it is possible to use this approach as a first step in identification of the orthologous target in these organisms (Collett et al., 2019).

Many drugs act by inhibiting essential enzymes, and thus depletion of the target by RNAi should be lethal, even in the absence of drug selection (Ullu et al., 2004). An exception to this involves knock down of a primary target that is a drug metabolising enzyme that converts a pro-drug into active drug metabolites, for example, nitroreductases (Baker et al., 2011; Hall and Wilkinson, 2012) or aldehyde dehydrogenase (Zhang et al., 2018).

**Figure 2:**
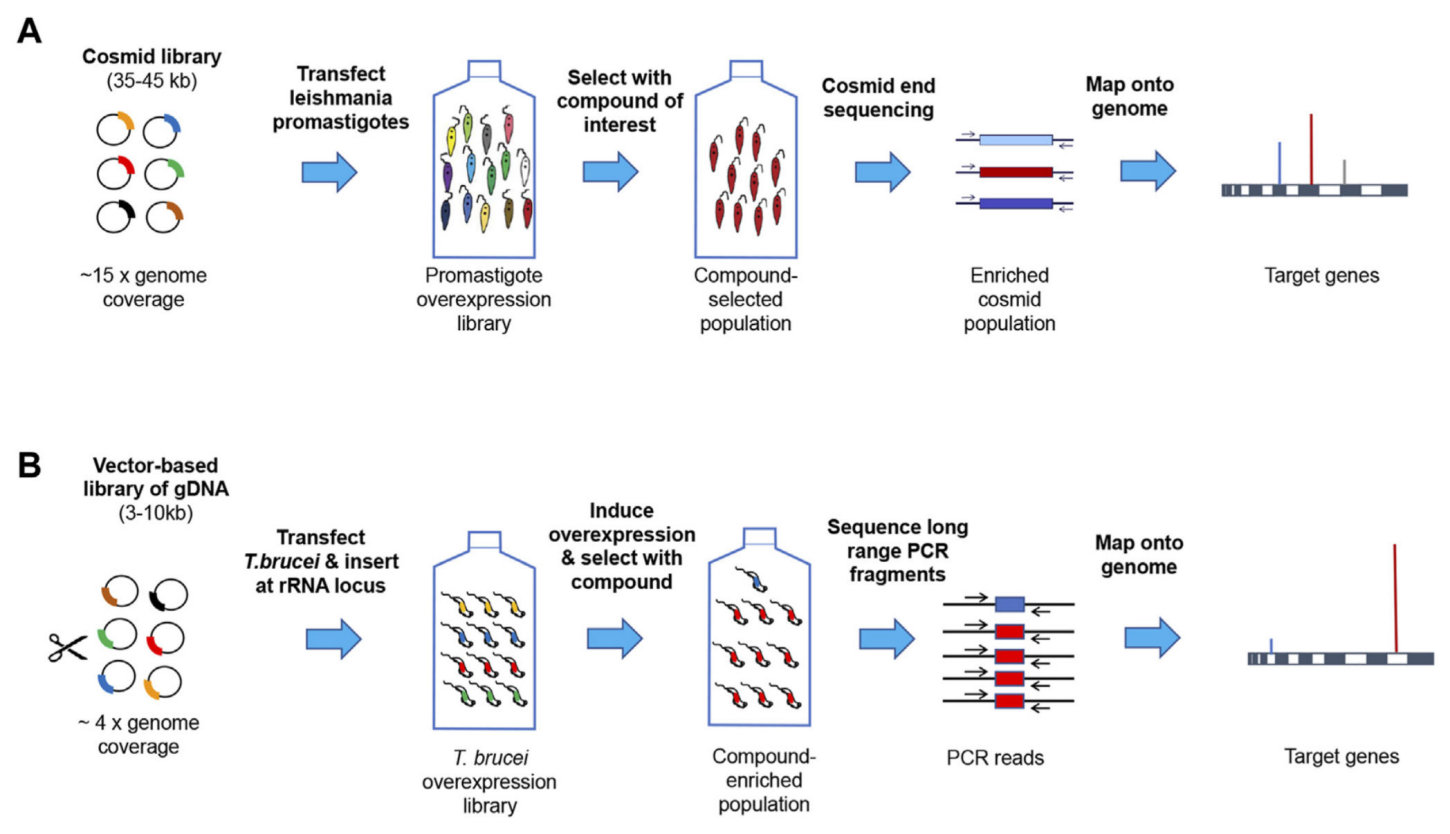
Genome-wide target overexpression strategies. Panel (**A**). Episomal cosmid expression strategy for Leishmania. Promastigotes are transfected with a shuttle cosmid vector pcosTL (Kelly et al., 1994) containing ~38 kb genomic DNA fragments under G418 selection (Hoyer et al., 2001). After the parasite cosmid library has undergone drug selection, cosmids are sequenced and mapped onto the reference genome (Corpas-Lopez et al., 2019). A similar approach is under development for *T. cruzi* at the University of Dundee. Panel (**B**). Expression from genes at the rRNA locus in *T. brucei*. Genomic DNA fragments were cloned in pRPaOEX, transfected into bloodstream form *T. brucei* and inserted at the rDNA locus under the control of a tetracycline-inducible ribosomal RNA (rRNA) promoter. Following drug selection, DNA was extracted from the surviving population and the overexpression inserts amplified by long range PCR, sequenced and mapped onto the reference genome (Corpas-Lopez et al., 2019).

In most cases target overexpression confers resistance to a compound’s toxic effect. This approach has been successfully applied in *T. brucei* (Begolo et al., 2014; Wall et al., 2018b), *L. donovani* (Ryan et al., 1993; Cotrim et al., 1999; Gazanion et al., 2016; Corpas-Lopez et al., 2019; Potvin et al., 2019; Paradela et al., 2021; Smith et al., 2022) and to a lesser extent in *T. cruzi* (Kelly et al., 1994; Taylor et al., 2011). The methods typically employ either episomal plasmid or cosmid expression vectors or use tetracycline-inducible expression at the ribosomal DNA locus, followed by whole genome sequencing and alignment to a reference genome (Figure 2).

**Figure 3:**
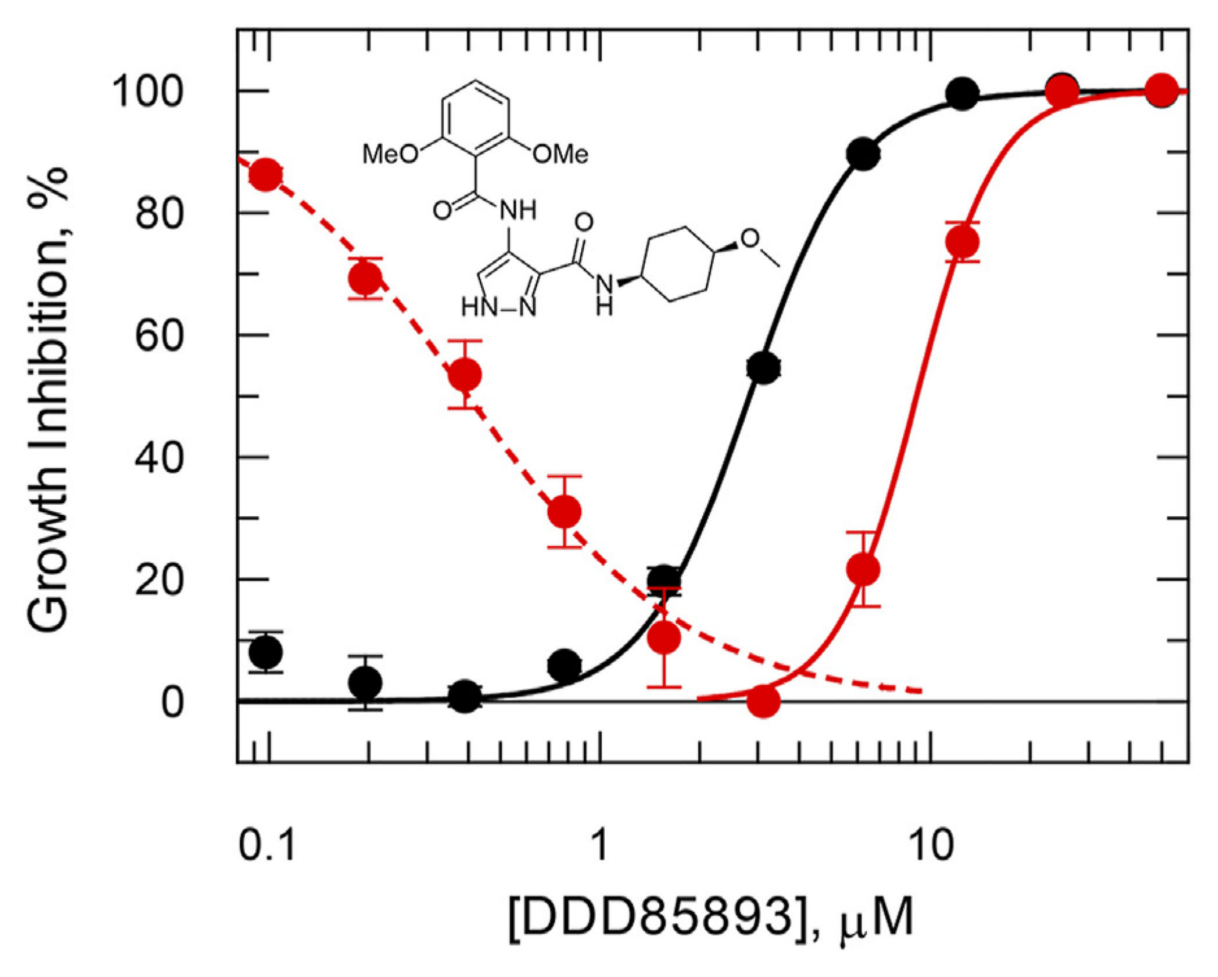
Rescued growth in a *T. brucei* cell line overexpressing GSK3β short. In the absence of tetracycline induction, cell growth (black symbols) follows a standard dose-response inhibition curve with an EC_50_ of 2.8 μM and slope 2.7. After tetracycline induction (red symbols) cell growth is stimulated up to maximum growth at 3 μM by DDD85893 (compound 4 m (Urich et al., 2014)) and inhibited thereafter with an EC_50_ of 9.1 μM and slope 3.6. Data redrawn from (Grimaldi, 2014). The dashed (growth stimulation) and solid (growth inhibition) lines are independent best non-linear fits to a dose-response equation at concentrations above and below 2 µM.

Overexpression of certain drug targets can sometimes lead to severe impairment of cell growth that can be restored by addition of an inhibitor. For example, knock down of glycogen synthase kinase 3 beta (GSK3β) short form by RNAi is associated with growth defects in *T. brucei* (Ojo et al., 2008; Alsford et al., 2011; Jones et al., 2014), and repeated failure to knock out both copies of the GSK3β gene, suggests essentiality (Grimaldi, 2014). In this case, it was important to demonstrate on-target activity against GSK3β in a cellular context, because medicinal chemistry was driven by cellular potency (Urich et al., 2014; Rojo et al., 2015) that can lead to off-target activity, particularly with protein kinase inhibitors (Woodland et al., 2013). Using a tightly regulated inducible overexpression system, overexpression of GSK3β was found to be toxic with decreased growth that could be “rescued” by increasing concentrations of DDD85893 up to 3 μM (Figure 3, dashed line). At concentrations above 3 μM, inhibition of growth is observed resulting in an overall decreased in potency of 2.5-fold (Figure 3, solid red line). Such findings provide strong evidence of on-target engagement by an inhibitor in a cellular context.

Generation of resistant clonal lines starting from a sensitive clonal line of parasites accompanied by whole genome sequencing is another powerful tool in target identification. This has been widely used in elucidating the targets and modes of action of drugs and experimental compounds in African trypanosomes and leishmania (Coelho et al., 2012; Jones et al., 2015; Wyllie et al., 2016a; Wyllie et al., 2018; Yasur-Landau et al., 2018; Bhattacharya et al., 2020; Hendrickx et al., 2021; Paradela et al., 2021; Rosa-Teijeiro et al., 2021; Alpizar-Sosa et al., 2022; Hefnawy et al., 2022). This approach involves stepwise exposure of wild-type kinetoplastid clonal lines to increasing (sublethal) drug concentrations over a period of weeks to months to obtain lines capable of proliferating normally in concentrations (generally >10 × EC_50_) that would be lethal for the original parental line (Jones et al., 2015; Wyllie et al., 2016a; Wyllie et al., 2018). After isolating clones from resistant cultures, the extent of resistance is determined, and stability of resistance assessed by sub-culturing in the absence of drug. Comparative whole genome sequencing of wild-type and resistant offspring can identify single nucleotide polymorphisms or copy number changes in specific genes, or gross chromosomal alterations associated with resistance. Genetic alterations observed in independent lines are more likely to be the target than other alterations present in only one clone. It should also be noted that identifying resistance-conferring mutations in kinetoplastids can be complicated by the fact that these are diploid organisms with heterozygous and homozygous mutations capable of modulating drug sensitivity. This approach also provides useful information on the speed with which, and the level of, resistance that can be obtained as well as the mechanisms of resistance such as uptake, efflux, metabolic inactivation and alterations to the level or affinity to a target (Fairlamb et al., 2016; Hefnawy et al., 2017). Recent studies by Bhattacharya and others have sought to expedite the generation of resistant *Leishmania* using a range of chemical mutagens (Bhattacharya et al., 2019). By combining chemical mutagenesis with next-generation sequencing, known as Mut-Seq, can lead to the rapid identification of mutations associated with drug resistance. However, it should be born in mind that not all resistance mechanisms are likely to arise readily in clinical situations due to drug pharmacodynamic behaviour *in vivo,* fitness costs and transmission potential associated with a resistance phenotype (Piel et al., 2018; Reis-Cunha et al., 2018; Van Bockstal et al., 2020).

### 2.2 Proteomics strategies

The potential of proteomics and chemo-proteomics approaches for target identification, validation, and identification of safety concerns in drug discovery has been the subject of some comprehensive reviews (Schenone et al., 2013; Ziegler et al., 2013; Boike et al., 2022; Meissner et al., 2022).

**Figure 4:**
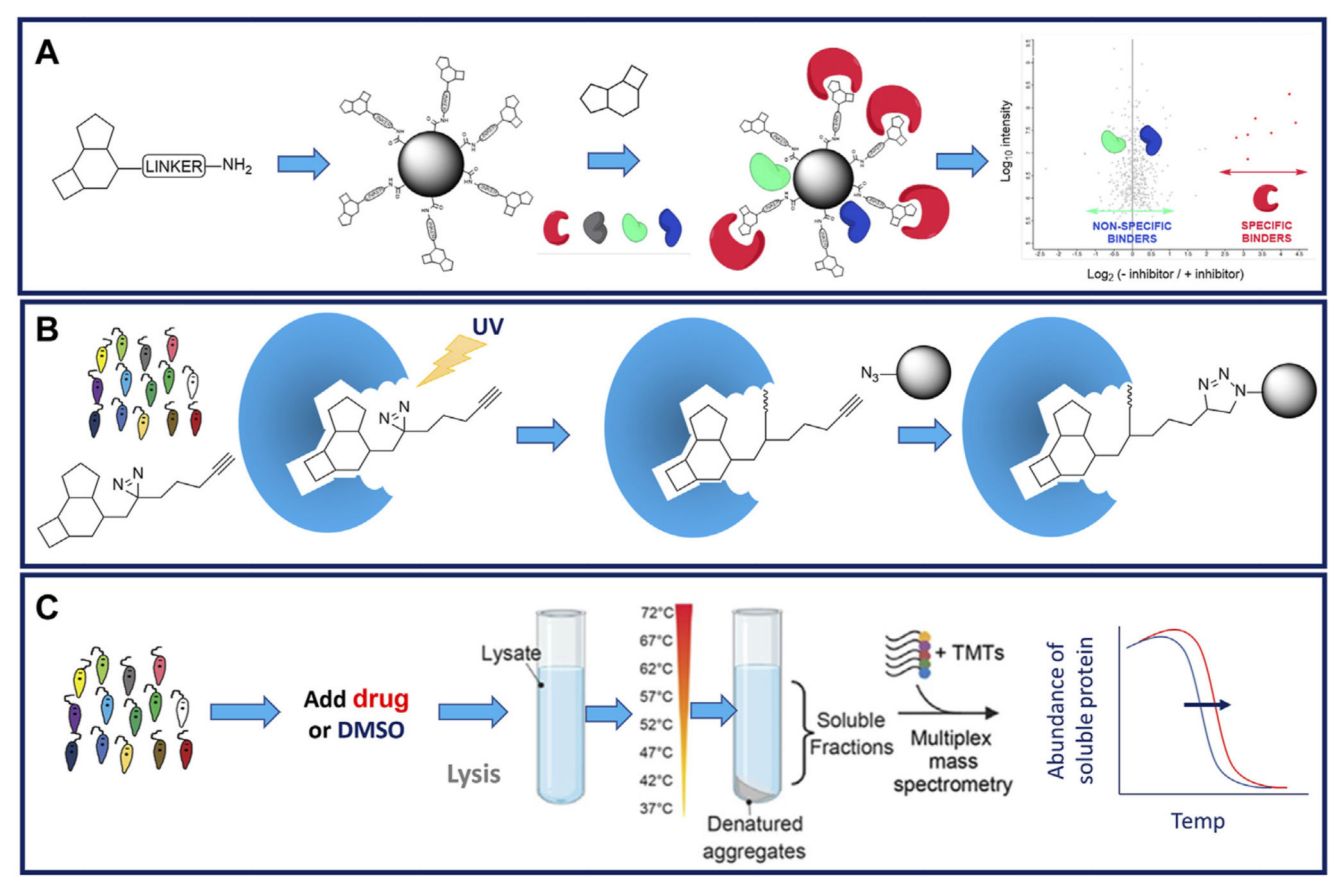
Schematic representation of proteomics approaches to drug target identification. Panel (**A**). Standard chemical pulldown workflow. Linkers (commonly PEG) are attached to a permissable position on the compound of interest. Linker analogues are attached to resin to create a “drug-bead”. Drug beads are incubated in parasite cell lysate that has been preincubated in the presence or absence of parent compound. Proteins binding to drug beads in the presence or absence of competition from the parent compound are identified by mass spectrometry. Proteins whose binding has been reduced in the presence of the parent compound are considered to be specifically binding to the linker compound and considered putative drug targets (Smith et al., 2023). Panel (**B**). Functionalised linkers comprising a diazarine photoactivatable warhead and an alkyne handle to facilitate subsequent pulldown are attached to a permissbale position on the compound of interest. These functionalised linker analogues are incubated with live cells. Covalent attachment of the linker analogue to its molecular target is triggered by exposure to UV light. Interacting targets are enriched from the cell lysates using the bio-orthogonal handle and identified by mass spectrometry. Panel (**C**). Standard thermal proteome profiling (TPP) workflow. Parasites are preincubated in the presence of drug or vehicle (DMSO). Treated and control cell lysates are prepared, aliquoted and aliqots incubated at a range of temperatures. Following incubation, soluble proteins are harvested, processed, labelled with TMTs, pooled and analysed by LC-MS/MS (Corpas-Lopez and Wyllie, 2021).

Chemical proteomics strategies can rightly be considered as the gold standard for drug target identification. This is principally due to the fact that, unlike many of the other approaches described in this review, chemical proteomics can provide evidence of compounds directly binding to their molecular target(s). In addition, these approaches can enable the affinity of the drug binding to its molecular target to be demonstrated. One such approach that is increasingly employed in target identification for kinetoplastids is chemical pulldown (Wyllie et al., 2018; Smith et al., 2022). Here, compounds of interest are immobilised onto a suitable resin to prepare “drug-beads” that are then used to pull down specifically binding molecular targets from parasite whole cell lysates (Figure 4A). Typically, immobilised ligands are linked through a polyethylene glycol (PEG) chain to biotin, or a suitable handle for covalent attachment to a resin (Ziegler et al., 2013). Biotin-labelled ligands can then be “immobilised” on streptavidin-coated beads to prepare an affinity resin. There are several alternative methods to covalently attach a linker-containing ligand to a resin: commonly the linker will have a terminal amine which will readily react with a resin containing an activated ester to form an amide (Ziegler et al., 2013). Note, the use of magnetic resins greatly simplifies the subsequent pull-down procedure.

It is important to ensure that the point of linker attachment to the ligand does not significantly reduce the phenotypic activity of the compound of interest (Meissner et al., 2022). In the worst-case scenario, a significant loss in potency can indicate that linker attachment has driven the compound of interest off-target. Therefore, it is essential to understand the structure-activity relationship (SAR) of any given inhibitor series in order to select suitable points for PEG linker attachment (Ziegler et al., 2013). The linker-functionalised ligands (or an advanced synthetic intermediate towards them) can be readily assayed to verify that they have exclusively retained the expected phenotype and potency. It is considered best practice to attach a linker to several different positions on the compound: if subsequent chemical proteomics experiments with two resins identify the same target proteins, then confidence in the results is increased (Smith et al., 2023).

One of the key challenges of chemical pulldowns is the presence of non-specific binding of proteins, particularly high abundance proteins, that can complicate the interpretation of experiments and/or prevent detection of proteins that bind specifically to the “drug bead”. This problem can be minimised by carrying out competition studies whereby parasite cell lysates are incubated with “drug beads” in either the presence, or absence of soluble inhibitor (Terstappen et al., 2007). Subsequent tryptic digestion of the resin-bound proteins followed by Tandem Mass Tag (TMT) labelling and mass spectrometry allows quantitative comparison between experiments (Bantscheff et al., 2011). Proteins that demonstrate reduced binding to drug beads in the presence of soluble inhibitor are considered specific binders and implicated in the ligand’s mechanism of action. Indeed, carrying out pulldowns in competition with a range of inhibitor concentrations can allow the affinity with which specific binders interact with the compound of interest (Wyllie et al., 2018).

The use of photoactivatable linkers (PAL) to facilitate chemical pulldowns, such as those pioneered by Cravatt and others (Hulce et al., 2013), has yet to be applied to drug target identification studies in kinetoplastids. PAL contain both a photo-affinity group (typically a diazirine) and a handle for bio-orthogonal chemistry (typically an alkyne) and are attached to compounds of interest at a permissible position. Live cells are incubated with PAL analogues and irradiated with UV light. UV exposure triggers the diazirine to form a covalent adduct with the molecular target. Interacting targets are enriched from the assay system using the bio-orthogonal handle and identified by mass spectrometry (Figure 4B). The major advantage of this approach, over standard chemical pulldown, is the ability to incubate the probe with live parasites rather than cell lysates thus enabling the probe to bind to its target in a physiologically relevant environment rather than a cell lysate. It is likely that PAL in their various iterations will be more deployed more broadly applied to target identification in these parasites in future.

The need to fully understand the SAR surrounding compounds of interest is perhaps the principal draw-back of chemical pulldown as an approach to support target identification, since this information may not be readily available for early phenotypic hits. An alternative, unbiased chemical proteomics strategy that does not require derivatization of the ligand and does not require knowledge of SAR is thermal proteome profiling (TPP) (Franken et al., 2015; Corpas-Lopez and Wyllie, 2021). This approach exploits the biophysical principle that binding of a ligand induces thermal stabilisation of target proteins. To monitor this phenomenon, compound-treated and control cells or cell lysate are exposed to a range of temperatures (Figure 4C). Soluble protein from each sample aliquot is harvested, labelled with TMTs to enable quantitation, and melting curves for each protein within the lysate is established by mass spectrometry. Proteins that exhibit a significant and reproducible shift in thermal stability in the presence of the inhibitor are short listed as potential molecular targets. Thus, TPP can be used as an effective and unbiased approach to demonstrate compound-target engagement and has been employed successfully in several studies with kinetoplastid parasites (Corpas-Lopez et al., 2019; Corpas-Lopez and Wyllie, 2021; Paradela et al., 2021; Lima et al., 2022). However, it should be noted that TPP is less amenable to the identification of protein targets that form part of large, stable protein complexes, such as the proteasome. The requirement for soluble cell lysate to support these studies also means that membrane protein targets are less likely to be identified.

Quantitative proteomics can also be used to directly compare protein expression profiles in drug-sensitive and resistant parasite cell lines. Differential labelling of wild-type and drug resistant proteomes can be achieved through Stable Isotope Labelling by/with Amino acids in Cell culture (SILAC) (Ong et al., 2002) or using TMTs. Following labelling, these samples can be combined and analysed via mass spectrometry. This approach was successfully employed to identify the enzyme involved in activating bicyclic nitroaromatic pro-drugs, including DNDI-VL-2098 (https://dndi.org/research-development/portfolio/vl-2098/), a candidate for visceral leishmaniasis, now abandoned due to testicular toxicity. Comparative proteomics revealed that *L. donovani* promastigotes resistant to DNDI-VL-2098 had lost a hypothetical NADH:FMN-dependent oxidoreductase (NTR2) subsequently confirmed to be responsible for pro-drug activation (Wyllie et al., 2016b).

### 2.3 Phenotype profiling

Observing the morphology of drug-treated parasites can illustrate the consequences or collateral damage inflicted by drug target inhibition; however, the jury is still out on the ability to use this information to identify the molecular targets of drugs or active compounds. For instance, *L. donovani* promastigotes exposed to an established proteasome inhibitor demonstrated a significant accumulation of intracellular vesicles, with the rationale that an inability to recycle key proteins via the proteasome left these parasites overcome by their own waste protein products (Wyllie et al., 2019). In addition, inhibition of the enzyme *N*-myristoyltransferase has been associated with the “big-eye” phenotype in African trypanosomes. This expansion of the parasite’s flagellar pocket was observed when the clathrin heavy chain (Rab5) (Allen et al., 2003; Hall et al., 2004) or Arf1 (Hall et al., 2004) were knocked down in *T. brucei* and is assumed to be due to the inhibition of endocytosis in these parasites. Soon, approaches such as cell painting (Bray et al., 2016) which uses multiplexed fluorescent dyes to profile morphological changes in drug-treated cells may be used to efficiently recognise previously deconvolved mechanisms of action. Similarly, cell cycle analysis by flow cytometry or other analogous approaches can be useful in profiling the broad mechanism of action of developmental compounds. However, akin to cell painting, this approach cannot be used to directly identify the molecular targets of phenotypic actives. Indeed, *L. donovani* promastigotes exposed to the proteasome inhibitor GSK3494245/DDD01305143 (Wyllie et al., 2018) and DDD853651/GSK3186899 (Wyllie et al., 2019), an inhibitor of the cyclin-dependent kinase CRK12, both arrest at the G2/M stage of the cell cycle. Thus, extrapolating such data to identify potential molecular targets of active compounds can be challenging.

### 2.4 Informatics approaches to facilitate drug target identification

Multiple *in silico* tools are now available to assist in predicting the biological targets of active compounds (Jenkins et al., 2006; Wang et al., 2013; Chen et al., 2016; Finan et al., 2017; Koscielny et al., 2017; Tanwar et al., 2022; Yu et al., 2022). These tools use data mining methods to exploit the wealth of data deposited in databases such as PubChem (Wang et al., 2009) and ChEMBL (Gaulton et al., 2012). Computational approaches such as chemical similarity searching, data mining/machine learning, bio-activity spectra, and panel docking can then be applied to link putative targets to compounds. Since these algorithms essentially learn from existing knowledge of compound—target pairs, at this stage they are far more effective in predicting human molecular targets (Yu et al., 2022). As discussed in this review, relatively few chemically validated targets have been identified in the kinetoplastids so far. This, combined with the evolutionary distance between these parasites and humans, is likely to limit the success of *in silico* target identification. It is hoped that concerted efforts to deconvolute the molecular targets of anti-kinetoplastid compounds can be used to improve the effectiveness of predictive tools. Nevertheless, putative targets identified through *in silico* approaches will always require direct validation within the specific parasites.

### 2.5 Metabolomic strategies

Metabolomics, or more correctly comprehensive metabolic profiling, in kinetoplastids has been the subject of recent reviews (Creek and Barrett, 2014; Vincent and Barrett, 2015; Fall et al., 2022). The general methodology involves extraction (with derivatization for certain metabolites), separation by ultraperformance liquid chromatography, liquid or gas chromatography, and detection by mass spectrometry or nuclear magnetic resonance. Metabolite identification remains a major bottleneck, particularly due to the wide dynamic range and chemical complexity of metabolic extracts. Nonetheless, comparative, targeted metabolomics offers a dynamic and precise picture of the drug-induced phenotype, providing insights into the mode of action of miltefosine (Vincent et al., 2014; Armitage et al., 2018a), antimonials (Berg et al., 2013; Rojo et al., 2015; Gutierrez Guarnizo et al., 2021), benznidazole (Trochine et al., 2014), suramin (Zoltner et al., 2020) and nifurtimox/eflornithine (Vincent et al., 2012). Metabolomics coupled with principal component analysis has been used to cluster hit compounds from the GSK *Leishmania* box (Peña et al., 2015) as to their potential mode of action, offering a novel screening approach for drug selection/prioritization (Armitage et al., 2018b). Metabolic profiling can also reveal unexpected secondary domino effects in mode of drug action. For example, the antifolate trimethoprim leads to direct inhibition not only of *E.coli* dihydrofolate reductase (its molecular target), but also indirect inhibition of folylpoly-γ-glutamate synthetase. Primary inhibition of dihydrofolate reductase leads to the predicted precursor-product relationship, where the increased dihydrofolate concentration directly inhibits folylpoly-γ-glutamate synthetase (Kwon et al., 2008).

## 3 Identification of undesirable targets and mechanisms of cell killing

The following examples are drawn from experience in the Drug Discovery Unit in Dundee on undesirable mechanisms that are unlikely to meet the desired therapeutic product profile for a particular disease indication (Nwaka and Hudson, 2006; Wyatt et al., 2011).

### 3.1 Generic chelators

A novel 7-substituted 8-hydroxy-1, 6-naphthyridine (8-HNT) series (Thomas et al., 2020) with promising activity against *T. brucei* and *L. donovani* emerged from screening a 1.8-million-compound library against *L. donovani* as part of a collaboration between GlaxoSmithKline (GSK) and the University of Dundee Drug Discovery Unit (Peña et al., 2015). Medicinal chemistry efforts struggled to markedly improve potency and selectivity, prompting an investigation into the mode of action of this compound series. Genome-wide knockdown (RNAi screens) revealed genes encoding a putative Golgi-localised zinc transporter decreased susceptibility, suggesting a role for divalent metal ions in the mode of action of these compounds (Wall et al., 2018a). Compounds from this series depleted intracellular Zn^2+^ and, conversely, exogenously added Zn^2+^ reduced the potency of the 8-HNT series. Spectrophotometric analysis demonstrated that these compounds bind directly to form a 2: 1 stoichiometric complex with either Zn^2+^, Cu^2+^ or Fe^2+^. Further work is required to establish if chelation of the latter divalent cations also plays a role in cytotoxicity. Given zinc’s broad role in enzyme catalysis (Andreini et al., 2009), protein structural stability (Cassandri et al., 2017) and redox biology (Oteiza, 2012), the identification of chelation as the main driver for cytotoxicity presents a significant challenge for further development. Consequently, work on this chemical series was abandoned in favour of more promising leads.

### 3.2 Cytochrome P450 lanosterol demethylase (CYP51)

Sterol metabolism in kinetoplastids involves both acquisition of cholesterol from the host and the *de novo* synthesis of ergosterol-related sterols (Roberts et al., 2003). The balance between these two options is driven by availability and ease of acquisition of preformed cholesterol from serum or cellular components (Coppens and Courtoy, 2000; Lepesheva et al., 2011; Lepesheva and Waterman, 2011; Nes et al., 2012). Despite strong chemical and genetic evidence of the essentiality and druggability of CYP51 in *T. cruzi in vitro* and in animal models, clinical trials with the antifungal posaconazole in Chagas’ disease patients have proven to be unsuccessful (Molina et al., 2014; Morillo et al., 2017). It appears that a minimum number of replications are required for posaconazole, a potent inhibitor of CYP51, to cause growth inhibition and that the resulting depletion of ergosterol content is not cytocidal in all parasites (MacLean et al., 2018). Consequently, the prevailing view is that CYP51 inhibitors have a low priority for further drug discovery efforts. As a result, a CYP51 assay has been introduced into our screening cascades to triage such undesirable hits (Riley et al., 2015) and a wash-out experimental design has been developed to distinguish cytocidal compounds from those that are cytostatic (MacLean et al., 2018).

### 3.3 Cytochrome *b* inhibitors

Trypanosomes and leishmania possess a single mitochondrion that is present either as a single tubule with few cristae (in bloodstream African trypanosomes) or a more complex reticulated network with plate-like cristae as in procyclic forms of *T. brucei* and all life cycle stages of *Leishmania* spp. and *T. cruzi*. A specialised region of the mitochondrion localised at the base of the parasite’s flagellum contains a large DNA structure—the kinetoplast (Shapiro and Englund, 1995). This is comprised of several thousand catenated small circular DNA (minicircles) and larger circular DNA (20–50 maxicircles). The maxicircles encode some thirteen proteins, including cytochrome *b* which is a key component of complex III of the electron transport chain, namely, cytochrome bc1 (quinol—cytochrome-c reductase, E.C. 7.1.1.8). The kinetoplastid mitochondrion is involved in the mode of action of a number of drugs and experimental compounds (Fidalgo and Gille, 2011), including the nitro-drugs nifurtimox and benznidazole (Wilkinson et al., 2008; Hall et al., 2011; Hall and Wilkinson, 2012), the diamidines pentamidine and DB analogues (Shapiro and Englund, 1990; Lanteri et al., 2008; Motta, 2008; Yang et al., 2016), and the antibiotics salicylhydroxamic acid (Clarkson and Brohn, 1976; Fairlamb et al., 1977) and ascofuranone (Yabu et al., 2003), that inhibit the cyanide-insensitive alternative oxidase present in bloodstream form African trypanosomes.

In *Leishmania*, naphthoquinones such as the antimalarial atovaquone and buparvaquone, used to treat cattle theileriosis, act on the cytochrome *bc1* complex blocking electron transport, inhibiting ATP synthesis and amastigote growth (Croft et al., 1992; Ortiz et al., 2016). A high throughput screen of 700,000 compounds at the Genomics Institute of the Novartis Research Foundation identified GNF7686 as an active growth inhibitor of *L. donovani* axenic amastigotes. GNF7686 was found to show promising activity against *T. cruzi* as well. Whole genome sequencing of drug-resistant and drug-sensitive clonal lines of *T. cruzi* identified a mutation (L198F) in the maxicircle gene encoding cytochrome *b* and biochemical studies confirmed the Q_N_ (Q_i_) site as the target of GNF7686 (Khare et al., 2015). A similar unbiased screen against *T. cruzi* and *L. donovani* by GSK (Peña et al., 2015) and subsequent lead optimisation identified three different chemotypes that were subsequently shown using a similar strategy to act in a comparable fashion inhibiting respiration by binding to complex III of the respiratory chain at the Q_i_ (Q_N_) site (Wall et al., 2020). Cytochrome *b* appears to be a promiscuous and readily druggable target with high potential for resistance (Wall et al., 2020); thus a counter screen using a cytochrome *b* resistant panel has been introduced into our drug development pipelines to prevent over-representation of such hits in our portfolio.

## 4 Targets of clinical candidate drugs

### 4.1 The proteasome

**Figure 5:**
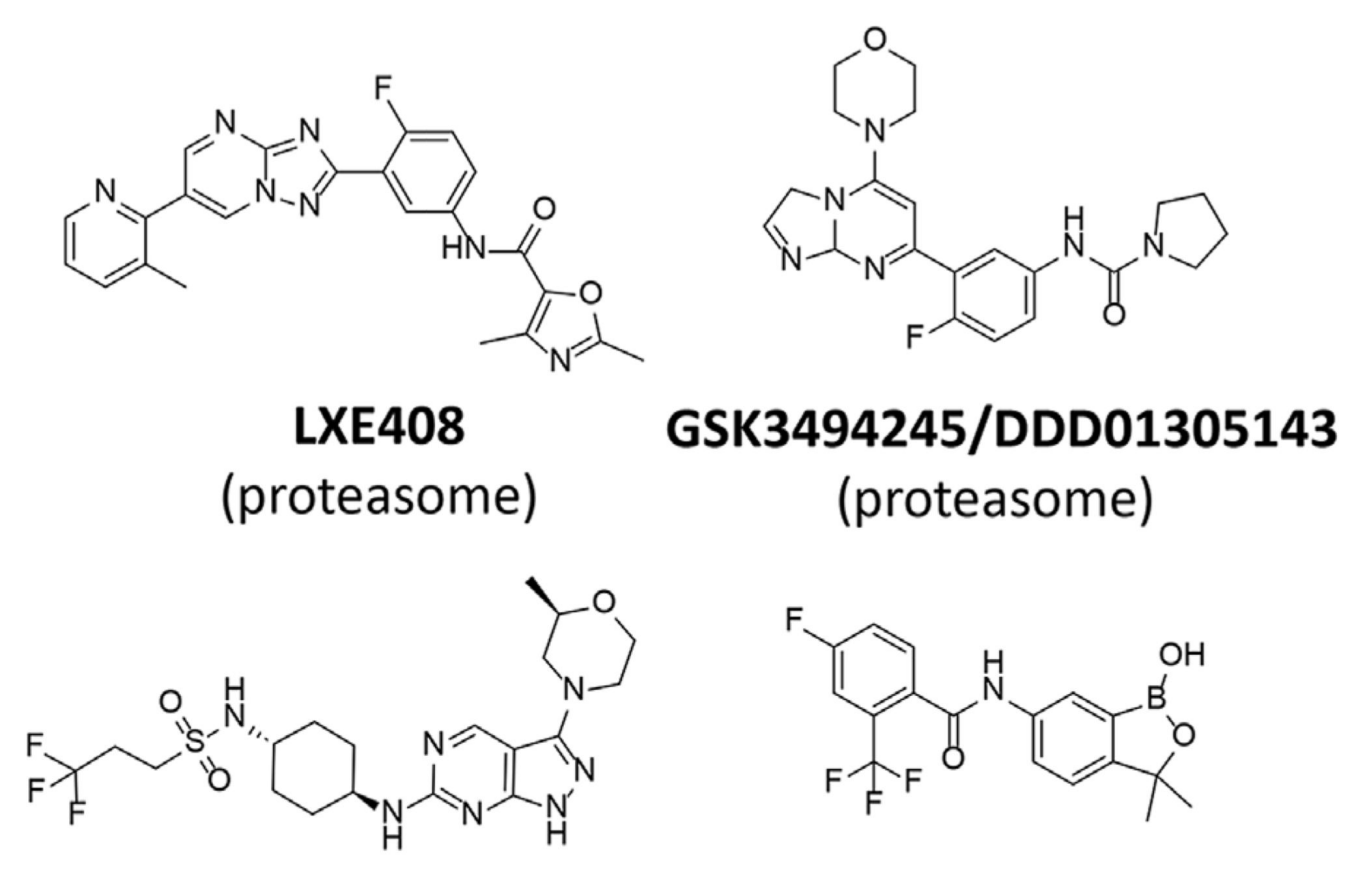
Structures and primary targets of clinical candidates.

Interest in the kinetoplastid proteasome (Hua et al., 1995) arose in the 1990 s as a result of the discovery and validation of ornithine decarboxylase (ODC) as the drug target for eflornithine (D, L, α-difluoromethylornithine) (Bacchi et al., 1980) for the treatment of Human African Trypanosomiasis (Van Nieuwenhove et al., 1985). Mammalian ODC is rapidly turned over in cells and degradation requires interaction with the polyamine-inducible protein antizyme and subsequent degradation by the proteasome (Li and Coffino, 1992; Murakami et al., 1992; Murakami et al., 1993). In contrast, *T. brucei* ODC is relatively stable. *T. brucei* lacks antizyme and differences between the trypanosomal and mammalian proteasome were proposed to account for the differential stability of *T. brucei* and mammalian ODC (Hua et al., 1995). Although various studies indicated that the proteasome played several essential roles in protein turnover and cell division in trypanosomes (Li and Wang, 2002) and *Leishmania* (Robertson, 1999; Paugam et al., 2003) there were no indications that this multi-subunit protease complex was selectively druggable until the discovery of a phenotypic hit (GNF5343) from a 3 million compound screen at Genomics Institute of the Novartis Research Foundation (Khare et al., 2016). Subsequent optimisation led to GNF6702 with efficacy in mouse models of visceral and cutaneous leishmaniasis, Chagas’ disease and stage 2 African trypanosomiasis. Prolonged exposure to compounds belonging to this series and whole genome sequencing identified a mutation (F24L) in the proteasome β4 subunit from *T. cruzi*. Subsequent biochemical experiments revealed that chymotrypsin-like activity of the *T. cruzi* proteasome was inhibited by GNF6702 (IC_50_ = 35 nM) and on-target activity was demonstrated from a tight SAR between the IC_50_ against the proteasome and EC_50_ against *L. donovani* axenic amastigotes and *T. brucei* bloodstream form trypanosomes. Optimisation of GNF6702 for improved solubility and favourable pharmacokinetic properties led to LXE408 (Figure 5) (Nagle et al., 2020) which is now in clinical development for the treatment of visceral leishmaniasis. A Phase I multiple ascending dose study of LXE408 was completed in September 2021 and the results of a Phase II, multicentre, randomized, two-arm blinded study to assess the efficacy and safety of LXE408 for treatment of visceral leishmaniasis are expected in 2025 (Drugs for Neglected Diseases initiative and Novartis Pharmaceuticals, 2022).

Phenotypic screening of a 15,659-compound diversity library against *T. cruzi* led to an initial hit that was subsequently identified in a second phenotypic screen as weakly active against amastigotes of *L. donovani* in macrophages. Optimisation for potency, selectivity, safety and other favourable pharmacological properties resulted in GSK3494245/DDD1305143 (Figure 5) being developed as a preclinical candidate for visceral leishmaniasis (Wyllie et al., 2019). Target knock-down with a genome-wide RNAi (RITseq) library in *T. brucei* identified 10 “hits” with functional domains commonly associated with proteins of the ubiquitin–proteasome recycling pathway. Highly resistant clones of *L. donovani* were generated and targeted sequencing of the genes encoding the β4 and β5 subunits of the proteasome revealed homozygous mutations in three independently generated resistant clones. Of these G197C of the β5 subunit was confirmed by genetic engineering to confer resistance. Chymotrypsin activity, but not trypsin or caspase activity, was inhibited by GSK3494245. Moreover, cell extracts from drug resistant lines were less sensitive to inhibition and cryo-electron microscopy of the related *L. tarentolae* proteasome revealed the binding site for GSK3494245 to lie between the β4 and β5 subunits (PDB 6QM7). Collectively, these and additional data provide strong evidence that disruption of proteasomal function is responsible for the cytocidal activity of GSK3494245.

### 4.2 Protein kinases

The preclinical candidate for visceral leishmaniasis, DDD853651/GSK3186899, has an interesting history. The original diaminothiazole hit was identified in a target-based screen against *T. brucei* GSKβ short form (Woodland et al., 2013). During lead optimisation cell potency did not track with target potency indicating that additional targets were drivers of trypanocidal activity. Lead compounds in this series also showed weak activity against axenic amastigotes of *L. donovani*. Replacement of the diaminothiazole core with a pyrazolopyrimindine core and multiple rounds of lead optimization in the “design, make, test, learn cycle” (Plowright et al., 2012) resulted in the pre-clinical candidate DDD853651/GSK318689 (Figure 5) (Wyllie et al., 2018; Thomas et al., 2019).

Three independent chemical proteomics approaches were used to identify the targets of this compound series (Wyllie et al., 2018). Several pyrazolopyrimidine analogues were used to pull down proteins in the presence or absence of a competitor compound using SILAC or isobaric tandem mass tag (ITRAQ) methodologies identifying 15 and 24 candidate targets, respectively. Common to both methods were cell division control-related kinases (CRK3, CRK6 and CRK12) and their cyclins (CYC3, CYC6 and CYC9). Drug treatment of cell cultures induced cell-cycle at arrest at the G1/S and G2/M phases consistent with a mode of action involving cell division control-related kinases. Quantitative mass spectrometry and Kinobead profiling of two pyrazolopyrimidine analogues in dose-dependent competition studies determined binding affinity to be in the rank order ~1 nM for CYC9/CRK12 and 25–100 nM for CYC3/CRK6.

Whole genome sequencing of drug-resistant clones of *L. donovani* identified many chromosomal and allelic changes. Notable among these were extra copies of chromosome 9 containing the CRK12 gene in 4 out of 6 clones with 3 of these 4 clones also having extra copies of chromosome 32 containing the likely partner cyclin CYC9. Co-overexpression of CRK12/CYC9 resulted in decreased sensitivity to drug, whereas a single knockout of CRK12 increased sensitivity. No mutations within, or amplification of, the CRK3 and CRK6 genes were found.

Collectively, these data point to protein kinase CRK12 and its partner cyclin CYC9 being the primary target for GSK3186899, although an element of polypharmacology involving other protein kinases cannot be discounted. Future studies include production of active leishmania CRK12/CYC9 complex and its structural determination, as well as elucidation of its functional role in parasites, including identification of its physiological substrates. Such information could guide second generation backups and identify potential novel drug targets. GSK3186899 is currently in Phase I clinical development by the Drugs for Neglected Diseases initiative (DND*i*).

Another promising protein kinase inhibitor series under development by Novartis is based on the aminobenzimidazole pharmacophore (Saldivia et al., 2020). This series has potent and selective pan-kinetoplastid activity *in vitro* against the kinetochore protein kinase CLK1. One compound in the series was effective in an acute model of African trypanosomiasis but failed to achieve cure in the CNS model. Screening of an inducible *T. brucei* library expressing individual protein kinases identified CLK1, a kinetochore component essential for mitosis, as a possible target. A unique feature of this series was the requirement of a Michael acceptor for potency that reacted to form a covalent bond with Cys215. Enzymatic, structural and cellular assays on mutants where this cysteine was replaced with either an alanine or serine residue demonstrated the importance of this amino acid for potency and selectivity over the human orthologue which has a serine at this position. Key challenges for the aminobenzimidazole series of compounds to progress to the clinic are to improve kinase selectivity and identify potent non-covalent inhibitors through structure-guided design (Saldivia et al., 2020).

### 4.3 Cleavage and polyadenylation specificity factor 3 (CPSF3)

The benzoxaborole class of compounds display wide anti-cancer, anti-fungal, anti-protozoal, anti-viral and anti-bacterial activity, as well as anti-inflammatory activity (Nocentini et al., 2018). Depending on the pharmacophore, oxaboroles and boronic acid drugs have modes of action involving: inhibition of the proteasome (bortezomib covalently interacts with the threonine catalytic residue in the chymotrypsin site (Groll et al., 2006)); carbonic anhydrases (coordination with the active-site zinc atom) (Nerella et al., 2022); and leucyl tRNA synthetases (covalent interaction with the cis diols of 3'-adenosine in tRNA^Leu^) (Seiradake et al., 2009; Hu et al., 2013; Sonoiki et al., 2016; Manickam et al., 2018; Si et al., 2019).

The anti-trypanosomal activity of benzoxaboroles was first discovered in a DNDi-sponsored phenotypic screen (Ding et al., 2010). Subsequent biological screening, medicinal chemistry and pharmacokinetic characterization identified SCYX-7158 (Figure 5) as an optimized analogue for stage 2 human African trypanosomiasis due to the drug’s favourable penetration of the CNS (Jacobs et al., 2011). Acoziborole (SCYX-7158 or AN5568) successfully completed Phase I human clinical trials in 2015 (NCT04270981) and recently completed Phase IIb/III for treatment for HAT (NCT03087955) (Betu Kumeso et al., 2022). In this open-label, non-comparative study, a single 960 mg oral dose of acoziborole was efficacious in 159 of 167 (95·2%) patients with late-stage gambiense HAT. The favourable safety profile and high efficacy of acoziborole should enhance efforts to reach the WHO goal of interrupting HAT transmission by 2030.

Understanding the trypanocidal targets of acoziborole would greatly inform the safety profile of this drug. Chemical proteomic profiling with an oxaborole-resin identified 13 enriched proteins including enzymes of RNA processing and glycolysis in wild-type *T. brucei* (Jones et al., 2015). Emergence of drug resistance against BSF *T. brucei* proved slow to develop and only moderate resistance was obtained by selection with a close structural analogue of acoziborole (Jones et al., 2015). Whole genome sequencing of three independently derived clonal lines revealed gross chromosomal copy number variants and single nucleotide polymorphisms. One clone showed 2-fold amplification of a short region of chromosome 4 that was also triploid, resulting in a 3-fold amplification of genes encoding CPSF3 and glyoxalase II. However, no single resistance determinant was found common to all three clones and Jones *et al* concluded that a degree of polypharmacology may be involved in the mode of action.

A second study using several benzoxaboroles observed inhibition of trans splicing of polycistronic mRNA as early as 1 hour after exposure to drug suggesting that mRNA processing could be a primary target of AN7973 (Begolo et al., 2018). Sensitivity to AN7973 in *T. brucei* was decreased three-fold by over-expression *T. brucei* CPSF3. AN7973 also caused metabolite changes indicative of disturbed methylation, similar to those observed for acoziborole. However, the lack of clear structure-function relationships for benzoxaboroles on trypanosome metabolites, or on splicing led Begolo *et al* to conclude that the modes of action of oxaboroles that target trypanosome mRNA processing might extend beyond CPSF3 inhibition.

In a third metabolomic study on the effects of acoziborole on *T. brucei*, significant perturbations in parasite metabolites were observed, particularly in *S*-adenosyl-L-methionine metabolism (Steketee et al., 2018). However, these changes may be a downstream consequence of inhibition of trans splicing since parasites were exposed to drug for an extended period.

In a fourth study using a high coverage cosmid overexpression library, Wall *et al* identified CPFS3 as the dominant “hit” accounting for 72% of all reads in the population that survived exposure to acoziborole and other benzoxaboroles (Wall et al., 2018b). These authors confirmed that overexpression of CPSF3 led to 4-fold decreased sensitivity to acoziborole. They also generated a homology model for molecular docking studies in which it was predicted that the oxaborole moiety would coordinate with the two zinc atoms in the active site. Selectivity over the human orthologue was attributed to steric hindrance at position 232 where the human has a bulky tyrosine moiety in place of an asparagine in the parasite enzyme. Attempts to edit an Asn232Tyr mutant was unsuccessful suggesting this change is not tolerated. Whilst these structural differences between trypanosome and host CPSF3 explain the safety profile and selective activity of acoziborole, definitive proof is lacking. Specifically, active recombinant protein and a suitable assay are required demonstrating binding to CPFS3 and selective inhibition of the parasite enzyme, ideally associated with structural evidence of ligand binding in the active site.

## 5 Discussion

Target identification and corresponding insight into the mode of action of a compound is of great value in accelerating drug discovery. Not only does it open opportunities for the development of target-based or pathway-based screens to identify alternative chemotypes as new start points for medicinal chemistry campaigns when an initial hit or lead series is failing to make progress, but also it can be used to initiate a structure-guided approach to improve potency and selectivity. In some cases, it may be possible to demonstrate engagement of an inhibitor with a molecular target in a whole cell context providing further reassurance that the correct strategy is in place. Knowledge of the target also alerts the drug discovery team to possible anti-targets in the patient, so that specific areas of host biology can be carefully monitored for possible undesirable effects. Examples include mitochondrial toxicity assays (cytochrome *b* inhibitors) or counterscreens against human orthologues such as a panel of protein kinases for CRK12 inhibitors, or a human RNA splicing assay and protein synthesis assay for benzoxaborole compounds. Such information can also be used to identify possible desirable—or eliminate likely undesirable—partner drugs for drug combination therapy. It can also be used as a portfolio management tool to ensure that a discovery programme is not overpopulated with compound series acting on the same target. Likewise, knowledge of a target or the mode of action of a compound series can deprioritise projects due to undesirable mechanisms, thereby diverting valuable resources of staff time and money to more promising projects.

From the examples given above, it should be clear to the reader that there is no single “one size fits all” approach to target identification. Indeed, the deployment of multiple orthologous approaches is the best strategy for target identification and provides greater confidence that the target and/or mode of action is driving the growth-inhibitory or cell death phenotype (Schenone et al., 2013). The notion that a drug exerts its action through modulation of a single target is not always the case, particularly with many older reactive drugs such as metalloids, polysulfonated or nitro-compounds and may also apply to reactive benzoxaboroles. As discussed here, multidimensional small-molecule profiling (Ortmayr et al., 2022) has accelerated the discovery of previously unknown targets in these parasitic organisms.

## Abbreviations

RNAi: RNA interference
SILAC: Stable isotope labelling by/with amino acids in cell culture
TPP: thermal proteome profiling
SAR: structure-activity relationship
PAL: photoactivatable linkers
DNDi: Drugs for neglected diseases initiative
TMT: tandem mass tag
